# Improvement of glucose and lipid metabolism via mung bean protein consumption: clinical trials of GLUCODIA™ isolated mung bean protein in the USA and Canada

**DOI:** 10.1017/jns.2017.68

**Published:** 2018-01-14

**Authors:** Mitsutaka Kohno, Hideo Sugano, Yuhko Shigihara, Yoshiaki Shiraishi, Takayasu Motoyama

**Affiliations:** 1 Fuji Oil Holdings Inc., R&D Division for Future Creation, 4-3 Kinunodai, Tsukubamirai, Ibaraki 300-2497, Japan; 2 J-PORT Company, 166 Georgetown Drive, Mountain View, CA 94043, USA

**Keywords:** Mung bean protein, Insulin, Homeostatic model assessment of insulin resistance, Serum TAG, Hepatic function enzymes, AE, adverse event, ALT, alanine aminotransferase, FPG, fasting plasma glucose, HOMA-IR, homeostatic model assessment of insulin resistance, MuPI, mung bean protein isolate, SPI, soya protein isolate

## Abstract

The aim of the present study was to confirm the effects of a commercially available mung bean protein isolate (GLUCODIA™) on glucose and lipid metabolism. The main component of GLUCODIA™ is 8S globulin, which constitutes 80 % of the total protein. The overall structure of this protein closely resembles soyabean β-conglycinin, which accounts for 20 % of total soya protein (soya protein isolate; SPI). Many physiological beneficial effects of β-conglycinin have been reported. GLUCODIA™ is expected to produce beneficial effects with fewer intakes than SPI. We conducted two independent double-blind, placebo-controlled clinical studies. In the first (preliminary dose decision trial) study, mung bean protein was shown to exert physiological beneficial effects when 3·0 g were ingested per d. In the second (main clinical trial) study, mung bean protein isolate did not lower plasma glucose levels, although the mean insulin level decreased with consumption of mung bean protein. The homeostatic model assessment of insulin resistance (HOMA-IR) values significantly decreased with mung bean protein. The mean TAG level significantly decreased with consumption of mung bean protein isolate. A significant increase in serum adiponectin levels and improvement in liver function enzymes were observed. These findings suggest that GLUCODIA™ could be useful in the prevention of insulin resistance and visceral fat accumulation, which are known to trigger the metabolic syndrome, and in the prevention of liver function decline.

Epidemiological reports from organisations such as the American Heart Association and the WHO have documented increasing obesity and diabetes epidemics^(^^1^^–^^4^^)^. The increase in the proportion of obese and diabetic individuals is significantly paralleled by an increase in the global incidence of the metabolic syndrome. In the most general sense, the metabolic syndrome is a cluster of risk factors occurring concurrently and increasing the risk for CVD, stroke and type 2 diabetes^(^^5^^)^. These risk factors include high blood pressure, high blood glucose level, obesity and lipid abnormalities, and are caused by visceral fat obesity and insulin resistance^(^^6^^)^.

There is considerable research assessing the efficacy of dietary nutrients in reducing the risk of the metabolic syndrome^(^^7^^)^. Of particular interest are protein isolates from soyabeans. The role of soya protein isolate (SPI) on insulin function and dyslipidaemia has been extensively studied^(^^8^^–^^10^^)^. For food labelling purposes, the US Food and Drug Administration has approved the health claim that the consumption of 25 g of SPI per d reduces the risk of heart disease^(^^11^^)^. However, a continuous consumption of 25 g of SPI is difficult to achieve by consumers because the volume is too large. It has been shown that β-conglycinin, which accounts for approximately 20 % of SPI, is a beneficial dietary nutrient for reducing the risk factors that cause diabetes, obesity and CVD. Therefore, β-conglycinin is regarded as the main component producing the beneficial physiological effects of SPI. Tachibana *et al*.^(^^12^^)^ studied normal adult rats consuming a diet containing β-conglycinin over 4 weeks, reporting decreased blood glucose and TAG levels and increased adiponectin levels. These changes were attributed to increased insulin sensitivity (i.e. improved glucose tolerance) of the liver. Several clinical trials have reported that continuous consumption of 2·5–5 g of β-conglycinin resulted in a reduction in visceral fat, serum TAG levels and lipid concentrations, and an increase in HDL-cholesterol concentration^(^^13^^–^^17^^)^. It is postulated that if highly purified β-conglycinin is efficiently and commercially isolated from soyabeans using food industrial methods, it would be possible to demonstrate the beneficial effects by continuous consumption of a small amount of protein. A number of simplified β-conglycinin isolation methods have been reported as being available in the food industry^(^^18^^–^^22^^)^. However, these methods are poor in practicality and have not yet been put to practical use.

The mung bean is a popular food crop in Asia, South America, Australia and the USA^(^^23^^)^. It is generally eaten as a boiled porridge, and mung bean starch is utilised in the form of noodles (bean-starch vermicelli), although the isolated protein itself is not typically consumed as a food. The main component of mung bean protein, accounting for over 80 % of the protein, is 8S globulin. 8S globulin has exhibited the highest degree of sequence identity (68 %) and structural similarity with β-conglycinin^(^^24^^)^. Mung bean protein isolate (MuPI) is expected to exhibit a four times stronger beneficial function on human health than SPI, in which β-conglycinin accounts for only 20 % of the total protein. 8S globulin was found to contain bioactive peptides that block angiotensin II-converting enzyme activity *in vitro* and was therefore hypothesised to alleviate hypertension^(^^25^^)^. An *in vivo* study in hamsters found that mung bean supplementation in their diets could decrease plasma cholesterol and TAG concentrations by up-regulating cholesterol-7α-hydroxylase (CYP7A1) mRNA and protein levels^(^^26^^)^. Of particular importance to the present study, GLUCODIA™, which is a commercially available MuPI produced as a by-product during starch production, was found to lower blood TAG levels in normal rats by inducing adiponectin and reducing TAG synthesis via insulin signalling^(^^27^^)^. In the present study, we examined the effect of GLUCODIA™ in a clinical trial setting and assessed its effects on metabolic syndrome-related factors.

## Experimental methods (used in both the pre-study and the main study)

### Experimental food

MuPI (GLUCODIA™) was manufactured using a method identical to that used for the production of SPI from the by-product containing the protein at the time of mung bean starch production and administered in the form of candy. GLUCODIA™, consisting of 92 % protein, 3 % minerals and 5 % water, was sterilised for food use and spray-dried. The composition of a test candy is shown in Table 1. In the control experiment, control candy with the same taste as the test candy was produced by replacing MuPI with the milk protein casein (Table 1). Therefore, in the case of the 6-g group, 4·4 g of 8S globulin were taken per d: 8S globulin (4·4 g) = GLUCODIA™ (6·0 g) × protein content (92 %) × 8S globulin content for total protein (80 %). So, the 3-g group consumed 2·2 g as 8S globulin, and the 1·5-g group consumed 1·1 g 8S globulin, respectively.

**Table 1.** Contents of chewable tablets

	Test chewable tablet			
Materials	6-g group	3-g group*	1·5-g group	Control chewable tablet*
GLUCODIA™ (mung bean protein) (%)	48·0	24·0	12·0	–
Milk protein (casein Na) (%)	–	24·0	36·0	48·0
Maltose (%)	34·5	34·5	34·5	34·5
Citric acid (%)	2·5	2·5	2·5	2·5
Flavour (%)	1·5	1·5	1·5	1·5
Lubricant (%)	2·5	2·5	2·5	2·5
Cellulose (%)	11·0	11·0	12·0	11·0
Total (%)	100	100	100	100

* The 3-g test and control chewable tablets were used in the pre- and main studies.

### Subjects

Test subjects were healthy men and women. Individuals undergoing treatment for hyperlipidaemia, diabetes, or liver dysfunction and those with food allergies were excluded from the study. Each study screened subjects based on health diagnostic results as shown in Table 2. Subjects were of non-child-bearing potential and included the following: women with a past history of hysterectomy, oophorectomy, or bilateral tubal ligation; post-menopausal (natural or surgically with >1 year since last menstruation) women; and women who agreed to use a medically approved method of birth control and who had a negative urine pregnancy test result. Subject recruitment was conducted at each research institution. The following recruitment methodologies were implemented to identify subjects for the study:

**Table 2.** Subjects and the outline of each study

	Pre-study	Main study
Subject selection criteria		
Age (years)	20–65	21–55
BMI (kg/m^2^)	25–40	25–35
FPG (mg/dl)*	110–125	100–125
2-h OGTT (mg/dl)*	140–200	–
TAG (mg/dl)†	–	120–300
Outline of test		
Test period (weeks)	4	8
Test group and	GLUCODIA™	GLUCODIA™ 3-g;
number of candy	1·5-g; 2 pieces	4 pieces
pieces consumed per	GLUCODIA™ 3-g;	
d	4 pieces	
	GLUCODIA™ 6-g;	
	8 pieces	
Number of subjects	8	25
(per group)		

FPG, fasting plasma glucose; OGTT, 2-h plasma glucose level using 75-g oral glucose tolerance test.

* To convert glucose from mg/dl to mmol/l, multiply by 0·0555.

† To convert TAG from mg/dl to mmol/l, multiply by 0·0113.

(A) Online recruitment: online advertising was ran almost daily throughout the study recruitment period.

(B) Database: a recruitment database was searched to identify potentially eligible subjects.

A participant withdrew from the study if it was determined by the qualified investigator that it was not in the participant's best interest to continue. This included adverse events (AE) or serious AE related to the investigational product causing clinically significant illness, the need for a prohibited concomitant medication, or a female participant who became pregnant during the course of the trial.

### Test design

Each study was a double-blind, placebo-controlled trial. The outline of the test design is shown in Table 2. The numbers of subjects who participated in the pre-study and main study were set with reference to the results of clinical studies on soyabean protein (β-conglycinin)^(^^28^^,^^29^^)^. The subjects in the test group were instructed to consume specified pieces of candy twice daily, before breakfast and dinner. Subjects in the control group were administered the casein candy in the same manner. The subjects recorded information, such as the presence or absence of consumption of the test candy, whether or not they took medications, alcohol consumption, and their physical state, in a diary from the start of the trial to the conclusion of the diet consumption period. Furthermore, the subjects recorded their diet and dietary content for the duration of the test period.

### Data collection

Data collection for each study was comparable except for body composition, which was assessed using dual-energy X-ray absorptiometry in the main study. Plasma samples were collected from each participant periodically and beginning at the start of the consumption period. Plasma samples were collected at 09.00 hours in the morning after fasting from 22.00 hours the previous night. The following serum biochemistry measurements were recorded: changes in fasting plasma glucose (FPG) and fasting insulin levels, lipid panels (total cholesterol, TAG, LDL-cholesterol, HDL-cholesterol, NEFA) and hepatic functional enzyme (aspartate aminotransferase, serum glutamic oxaloacetic transaminase, alanine aminotransferase (ALT), serum glutamic pyruvic transaminase and γ-glutamyltransferase) levels. The homeostatic model assessment of insulin resistance (HOMA-IR) was calculated as follows, based on each subject's FPG and fasting insulin levels: HOMA-IR = fasting insulin level (μU/ml) × FPG level (mg/dl)/405 (HOMA-IR = fasting insulin level (μU/ml) × FPG level (mmol/l)/22·5). Adiponectin level was measured only in the main study. The study safety indicators included the following: changes in blood pressure and heart rate, and blood safety parameters (complete blood count and comprehensive metabolic panel results: blood urea N, creatinine, total protein, albumin, globulin, total bilirubin, Ca, Cl, Na and K levels). The frequency and severity of AE and serious AE were recorded in detail, based on information obtained from interviews during each visit.

In the data analysis of the main study, data of subjects with HOMA 1·7 or more were analysed as low insulin sensitivity subjects, as well as analysis of all subjects except those who dropped out.

### Statistical analysis

Paired *t* tests were used to assess within-group changes from baseline to each time point in all studies. Comparisons among product groups were assessed by one-way ANOVA (pre-study), and Student's unpaired *t* test was used to assess differences between the groups at the end time of the test (main study). Data in Tables 3–5 are presented as means and standard deviations and a significance level (two-tailed) of <0·05 was considered to indicate a significant difference. A significance level (two-tailed) between 0·05 and 0·10 was set as a statistically significant tendency.

**Table 3.** Characteristics and plasma levels of pre-study subjects (Mean values and standard deviations; numbers of subjects)

		6-g group		3-g group		1·5-g group		Control	
		Mean	sd	Mean	sd	Mean	sd	Mean	sd
Subjects (*n*)		7		6		8		8	
Male		5		4		4		4	
Female		2		2		4		4	
Age (years)		40·0	13·7	40·5	9·1	42·9	8·2	44·9	13·6
Height (cm)		174·6	9·8	164·2	5·9	172·2	8·2	169·4	9·9
Weight (kg)		88·8	18·8	86·8	14·7	88·1	14·4	89·0	13·3
Ethnicity (*n*)									
White		3		2		3		2	
Hispanic		2		2		2		3	
African-American		1		1		1		1	
Asian		0		1		0		1	
American Indian/Alaska Native		0		0		1		0	
Native Hawaian/Pacific Islander		0		0		0		0	
Other		1		0		1		1	
FPG (mg/dl)§	Baseline	92·2	8·7	89·3	4·9	87·7	4·8	92·3	6·5
Week 4	87·5*‡	7·4	86·3‡	10·5	88·9	6·8	95·1	7·6
Δ FPG (mg/dl)§		−4·7†	6·1	−3·0†	3·9	1·1	6·5	2·9	5·1
Insulin (µU/ml)	Baseline	10·4	9·3	13·2†	0·5	9·1	4·8	8·9	4·8
Week 4	6·8	2·6	11·7	11·3	8·8	4·8	10·0	6·2
Δ Insulin (µU/ml)		−3·6	7·7	−1·6	11·5	−0·3	1·7	1·2	2·0
HOMA-IR	Baseline	2·4	2·1	2·9‡	0·2	2·0	1·1	2·0	1·1
Week 4	1·4	0·5	2·5	2·4	2·0	1·1	2·4	1·7
Δ HOMA-IR		−0·9‡	1·8	−0·4	2·4	0·0	0·6	0·4	0·6
TAG (mg/dl)ǁ	Baseline	103	39	125	34	102	29	97	48
Week 4	95	53	96‡	27	109	42	99	29
Δ TAG (mg/dl)ǁ		−9	50	−29‡	19	8	46	2	32
BMI (kg/m^2^)	Baseline	29·1	3·8	32·2	4·8	29·7	10·1	31·0	6·2
Week 4	28·9*†	1·0	32·0*	4·4	29·9	12·3	31·0	6·4
Δ BMI (kg/m^2^)		−0·2	0·2	−0·3	0·2	0·3	0·8	0·0	0·7

FPG, fasting plasma glucose; HOMA-IR, homeostatic model assessment of insulin resistance.

* Significant difference between baseline and week 4 data (*P* < 0·05).

† Significant difference between test and control data (*P* < 0·05).

‡ Significant tendency between test and control data (*P* < 0·1).

§ To convert glucose from mg/dl to mmol/l, multiply by 0·0555.

ǁ To convert TAG from mg/dl to mmol/l, multiply by 0·0113.

**Table 4.** Initial and final data of clinical characteristics and plasma levels of main-study subjects (Mean values and standard deviations; numbers of subjects)

Group…	Test: GLUCODIA™				Control: casein			
Consumption period (week)…	0		8		0		8	
	Mean	sd	Mean	sd	Mean	sd	Mean	sd
Subjects (*n*)	22				22			
Male	10				9			
Female	12				13			
Age (years)								
Mean	41·6				42·8			
sd	1·8				1·8			
Ethnicity (*n*)								
White	15				19			
Hispanic	3				1			
African-American	0				0			
Asian	2				2			
American Indian/Alaska Native	2				0			
Native Hawaian/Pacific Islander	0				0			
Other	0				0			
Weight (kg)	86·5	10·3	83·3	20·0	92·4	11·7	90·4	12·6
BMI (kg/m^2^)	30·5	3·0	31·0	3·3	30·7	2·9	30·6	3·0
Body fat ratio (%)	40·6	6·7	40·1	7·7	37·5	9·4	37·3	10·8
Fat body mass (g)	33 772	6446	33 509	7588	33 304	9197	33 057	10 853
Lean body mass (g)	49 616	8795	50 132	9465	55 565	11 059	55 418	11 605
Fasting plasma glucose (mg/dl)§	102	9	99	10	97	8	98	12
Insulin (μU/ml)	14·9	6·0	10·8**	4·7	12·2	4·8	13·7	7·6
HOMA-IR	3·8	1·8	2·9**	1·9	2·8	1·2	3·3	2·5
HbA1c (%)	5·59	0·31	5·59	0·28	5·50	0·27	5·45	0·23
TAG (mg/dl)ǁ	153	70	142‡	70	136	45	146	48
Total cholesterol (mg/dl)¶	197	37	190	40	197	22	195	31
LDL-cholesterol (mg/dl)¶	122	33	114	31	119	21	119	23
HDL-cholesterol (mg/dl)¶	50	12	47*	14	51	9	48**	9
NEFA (mEq/l)	530	311	460	213	383	199	428	238
AST (IU/l)	24·2	11·1	24·7	5·7	26·2	6·8	25·8	7·8
ALT (IU/l)	35·3	15·1	30·6*	13·6	31·0	12·1	31·6	14·8
γ-GTP (IU/l)	29·2	19·8	28·9	17·6	26·2	27·8	26·2	21·3
Adiponectin (μg/ml)	8·31	4·90	13·52**	9·19	13·09†	5·82	11·44	5·48

HOMA-IR, homeostatic model assessment of insulin resistance; HbA1c, glycated Hb; AST, aspartate aminotransferase; ALT, alanine aminotransferase; γ-GTP, γ-glutamyltransferase.

Significant difference between baseline and 8-week data: * *P* < 0·05, ** *P* < 0·01.

† Significant difference between test and control data (*P* < 0·05).

‡ Significant tendency between test and control data (*P* < 0·1).

§ To convert glucose from mg/dl to mmol/l, multiply by 0·0555.

ǁ To convert TAG from mg/dl to mmol/l, multiply by 0·0113.

¶ To convert cholesterol from mg/dl to mmol/l, multiply by 0·0259.

**Table 5.** Initial data of plasma levels of low insulin sensitivity subjects (homeostatic model assessment of insulin resistance (HOMA-IR) ≥ 1·7) in the main study (Mean values and standard deviations; numbers of subjects)

	Test: GLUCODIA™		Control: casein	
	Mean	sd	Mean	sd
Subjects (*n*)	20		16	
Male	10		6	
Female	10		10	
Fasting plasma glucose (mg/dl)*	102	9	98	8
Insulin (μU/ml)	15·2	5·7	13·7	3·9
HOMA-IR	3·9	1·5	3·3	1·0
HbA1c (%)	5·66	0·26	5·49	0·25
TAG (mg/dl)†	146	69	132	48
Total cholesterol (mg/dl)‡	195	37	193	23
LDL-cholesterol (mg/dl)‡	120	33	117	21
HDL-cholesterol (mg/dl)‡	51	12	50	9
NEFA (mEq/l)	539	323	398	188
AST (IU/l)	24·4	10·1	26·5	6·3
ALT (IU/l)	36·1	14·3	33·9	11·4
γ-GTP (IU/l)	28·3	18·3	29·8	28·0

HbA1c, glycated Hb; AST, aspartate aminotransferase; ALT, alanine aminotransferase; γ-GTP, γ-glutamyltransferase.

* To convert glucose from mg/dl to mmol/l, multiply by 0·0555.

† To convert TAG from mg/dl to mmol/l, multiply by 0·0113.

‡ To convert cholesterol from mg/dl to mmol/l, multiply by 0·0259.

### Adverse events

An AE was defined as any untoward medical occurrence in a clinical investigation participant who was administered an investigational product which did not necessarily have a causal relationship with the product. An AE could be any unfavourable and unintended sign (including an abnormal laboratory finding), symptom, or disease temporally associated with the use of a product, whether or not it was considered related to that product. Pre-existing conditions which worsened during the study were reported as AE. During the study, participants recorded AE in their diary. At each visit the participant was asked ‘Have you experienced any difficulties or problems since I saw you last?’. Any AE were documented in the study record and were classified according to the description, duration, intensity, frequency and outcome. The qualified investigator assessed any AE and decided causality. Intensity of AE was graded on a three-point scale (mild, awareness of event but easily tolerated; moderate, discomfort enough to cause some interference with usual activity; severe, inability to carry out usual activity), and reported in detail in the study record. The causality relationship of the investigational product to the AE was assessed by the qualified investigator as either:

Most probable: There was a reasonable relationship between the investigational product and the AE. The event responded to withdrawal of investigational product (dechallenge) and recurred with rechallenge when clinically feasible.

Probable: There was a reasonable relationship between the investigational product and the AE. The event responded to dechallenge.

Possible: There was a reasonable relationship between the investigational product and the AE. Dechallenge information is lacking or unclear.

Unlikely: There was a temporal relationship to investigational product administration but there was no reasonable causal relationship between the investigational product and the AE.

Not related: No temporal relationship to administration of the investigational product or there was a reasonable causal relationship between non-investigational product, concurrent disease or circumstance and the AE.

### Ethical considerations

Each study was conducted according to the guidelines set out in the Declaration of Helsinki, and written informed consent was obtained from all subjects/patients. Informed consent was obtained from each participant at the screening visit prior to any study-related activities being performed. Institutional review board approval of the protocol of pre-study was granted on 22 October 2013 by the Western Institutional Review Board (WIRB, study number: 1134705, WIRB PRO NUM: 20121473, INVEST NUM: 160744). All recruitment materials were approved by the institutional review board prior to use. The main study was reviewed by the Natural Health Products Directorate (NHPD), Health Canada and the research ethics board. Notice of authorisation was granted on 10 December 2014 by the NHPD, Ottawa, Ontario and unconditional approval was granted on 6 January 2015 by the institutional review board (IRB Services, Aurora, Ontario). The institutional review boards of IRB Services are registered with the Office for Human Research Protection (OHRP) and the Food and Drug Administration (ON IRB registration no. IRB00000766 and QC IRB registration no. IRB00005920).

## Results

### Pre-study

#### Subjects

The characteristics of the subjects are shown in Table 3. During the evaluation of efficacy, data from three subjects markedly reduced the accuracy of that evaluation; therefore, the three subjects were excluded from the analysis. The reasons for exclusion were: (1) non-compliance with failure to consume the study product (*n* 2); and (2) lost to follow-up (*n* 1). Finally, data from twenty-nine subjects were analysed.

#### Evaluation of safety

There were no statistically significant differences in blood pressure, heart rate and the blood safety parameters within each group and between the test and control groups at any time point. The frequency and intensity of AE were established from interviews occurring during each visit. One subject complained of psychological AE (irritability, impatience and fatigue), and two subjects complained of gastrointestinal AE (diarrhoea, abdominal cramping, bloating and nausea). By the qualified investigator, intensities of all observed AE were classified as ‘mild’ on a three-point scale, and the causality relationships of the investigational product to these AE were assessed as ‘not related’.

#### Fasting plasma glucose, insulin and homeostatic model assessment of insulin resistance results

Mean FPG, insulin and HOMA-IR are shown in Table 3. The FPG levels in the 6-g and 3-g groups decreased during the study period. There were significant differences between the net changes in FPG level in the 6-g and control groups, and between the 3-g and control groups, respectively (*P* < 0·05, for both). The insulin levels in the test groups decreased dose-dependently. However, the net changes did not differ significantly between the test and control groups. Similarly, the HOMA-IR values in the test groups showed a dose-dependent decrease (Table 3). There was a significant tendency in the net change in HOMA-IR values between the 6-g and the control groups (*P* < 0·1).

#### TAG and BMI

The initial TAG level in the 3-g group was slightly higher (125 (sd 34) mg/dl; 1·41 (sd 0·38) mmol/l) than those in other groups. In this group, the TAG level decreased to 96 (sd 27) mg/dl (1·08 (sd 0·31) mmol/l) significantly. There was a significant tendency in the difference in TAG levels between the 3-g and control groups (Table 3, *P* < 0·1). BMI significantly decreased in the 6-g and 3-g groups between the test periods (*P* < 0·05; both groups).

### Main study

#### Subjects

**Fig. 1.**
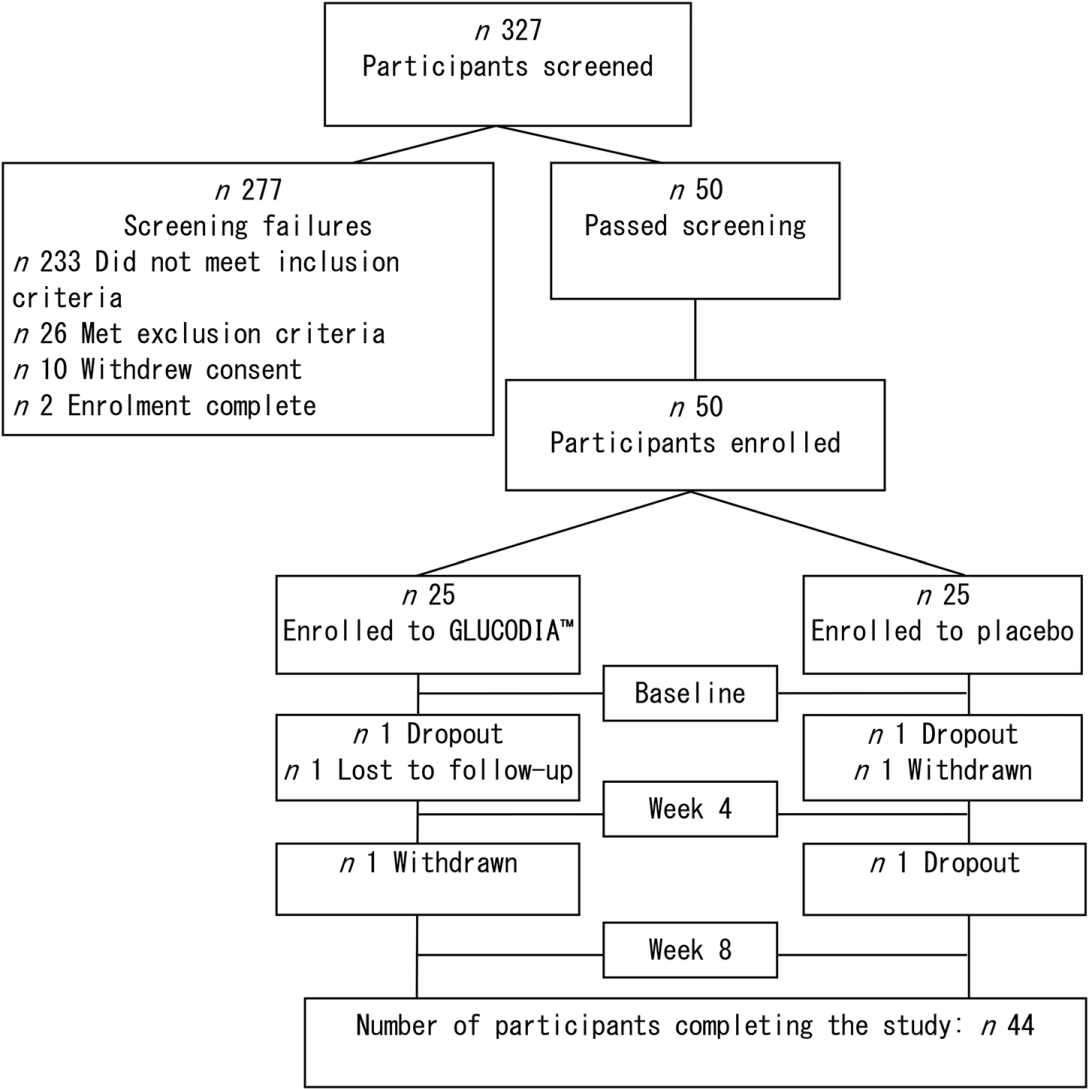
Dispositions of the main study participants. A total of 327 potential participants were screened, and fifty eligible participants were enrolled in the study. Forty-four participants completed the study.

Six subjects were removed from the per-protocol analysis (Fig. 1) for the following reasons: three subjects did not complete the study (continuous intakes of the test meal were refused); one subject was lost to follow-up; one subject withdrew consent from the study; and one subject was withdrawn due to poor compliance with the study protocol.

The background information of the forty-four subjects in both groups is summarised in Table 4. The FPG values in the test and control groups were 102 (sd 9) and 97 (sd 8) mg/dl (5·7 (sd 0·5) and 5·4 (sd 0·4) mmol/l), respectively; the insulin values were 14·9 (sd 6·0) and 12·2 (sd 4·8) µU/ml, respectively. There were no significant differences in the initial FPG and insulin levels between the test and control groups. BMI values in the test and control groups were 30·5 (sd 3·0) and 30·7 (sd 2·9) kg/m^2^, respectively; body fat ratios were 40·6 (sd 6·7) and 37·5 (sd 9·4) %, respectively. There were no significant differences in the initial BMI and body fat ratio between the two groups. Adiponectin values in the test and control groups were 8·31 (sd 4·90) and 13·09 (sd 5·28) µg/ml, respectively. There was a significant difference in the initial adiponectin level between the test and control groups (*P* < 0·05).

#### Evaluation of safety

There were no significant differences in the haematological and clinical chemistry parameters between the two groups. Differences in the following variables were significant within the test group (corpuscular Hb, 29·6 (sd 6·6) to 29·3 (sd 8·0) pg, *P* < 0·05; corpuscular Hb concentration, 340 (sd 33) to 338 (sd 42) g/l, *P* < 0·05; urea, 5·2 (sd 1·1) to 5·6 (sd 1·4) mmol/l, *P* < 0·05). In the control group, differences in the following variables were significant: corpuscular volume (88·1 (sd 14·1) to 87·8 (sd 12·2) fl; *P* < 0·05), corpuscular Hb (30·3 (sd 5·2) to 29·9 (sd 5·1) pg; *P* < 0·01) and Na (141·3 (sd 9·4) to 140·0 (sd 6·1) mmol/l; *P* < 0·05). However, these changes were within the normal clinical ranges and were not of clinical significance. A total of eight AE were reported, of which five occurred in the test group and three in the control group. The AE were experienced by five subjects: five AE were reported by two subjects in the test group, and three AE were reported by three subjects in the control group. The five AE in the test group were: dyspepsia (*n* 2), infrequent bowel movements (*n* 1) (both gastrointestinal disorders), headache (*n* 1) and thirst (*n* 1); the three AE in the control group were: nausea (*n* 1), upper abdominal pain (*n* 1) (both gastrointestinal disorders) and headache (*n* 1). By the qualified investigator, intensities of all observed AE were classified as ‘mild’ on a three-point scale, and the causality relationships of the investigational product to these AE were assessed as ‘unlikely’ or ‘not related’, respectively.

#### Fasting plasma glucose, insulin and homeostatic model assessment of insulin resistance (all subjects)

The mean FPG and insulin test results are shown in Table 4. The FPG levels in the test and control groups did not change during the meal consumption period. Fasting insulin levels in the test group showed a significant decrease from 14·9 (sd 6·0) to 10·8 (sd 4·7) µU/ml (*P* < 0·01). In contrast, fasting insulin levels in the control group showed a slight increase. The HOMA-IR values in the test group showed a significant decrease tendency, from 3·8 (sd 1·8) to 2·9 (sd 1·9) (*P* < 0·01), while these values increased in the control group (Table 4).

#### Lipid panel (all subjects)

TAG levels in the test group showed a significant decrease tendency from 153 (sd 70) to 142 (sd 70) mg/dl (1·73 (sd 0·79) to 1·60 (sd 0·79) mmol/l) (*P* < 0·1). Conversely, TAG levels in the control group slightly increased (Table 4). The total cholesterol and LDL-cholesterol levels in the test and control groups did not change during the meal consumption period. HDL-cholesterol levels in the two groups showed a significant decrease (test group, *P* < 0·05; control group, *P* < 0·01, Table 4). NEFA levels decreased in the test group and increased in the control group.

#### Hepatic functional enzymes (all subjects)

Aspartate aminotransferase and γ-glutamyltransferase levels in the test and control groups did not change during the meal consumption period. ALT levels in the test group significantly decreased from 35·3 (sd 15·1) to 30·6 (sd 13·6) IU/l (*P* < 0·05). Conversely, ALT levels increased slightly in the control group (Table 4).

#### Adiponectin (all subjects)

The adiponectin level in the test group significantly increased (*P* < 0·01) and that in the control group decreased (*P* = 0·107). The net changes in adiponectin levels in the test and control groups were 5·20 (sd 8·44) and −1·65 (sd 5·35) µg/ml, respectively. There was a significant difference between the net changes in the test and control groups (*P* < 0·01).

#### Background information on participants with low insulin sensitivity

Subjects with HOMA-IR ≥ 1·7, as mild or above insulin resistance subjects, were selected^(^^30^^)^. The background information on these subjects is shown in Table 5.

#### Fasting plasma glucose, insulin and homeostatic model assessment of insulin resistance (participants with low insulin sensitivity)

**Fig. 2.**
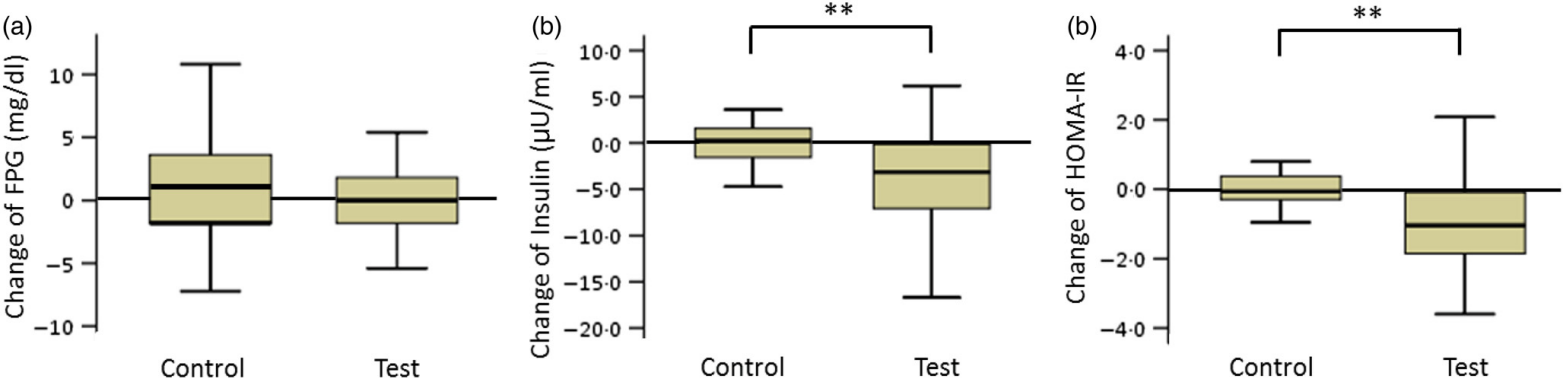
Box plots of net changes in the parameters of glucose metabolism among participants with low insulin sensitivity: (a) fasting plasma glucose (FPG); (b) insulin; (c) homeostasis model assessment of insulin resistance (HOMA-IR). Whiskers represent the minimum and maximum values, and the boxes represent the lower and upper quartiles. ** Significant difference between the test (GLUCODA™) and control groups (*P* < 0·01). To convert glucose from mg/dl to mmol/l, multiply by 0·0555.

The FPG levels in the test and control groups did not change during the consumption period. The net changes in insulin levels and HOMA-IR values in the test and control groups were −4·1 (sd 6·1) and 1·8 (sd 5·1) µU/ml (insulin) and −1·0 (sd 1·9) and 0·6 (sd 1·8) (HOMA-IR), respectively. These net changes were significantly different (*P* < 0·01; Fig. 2).

#### Lipid panel (participants with low insulin sensitivity)

**Fig. 3.**
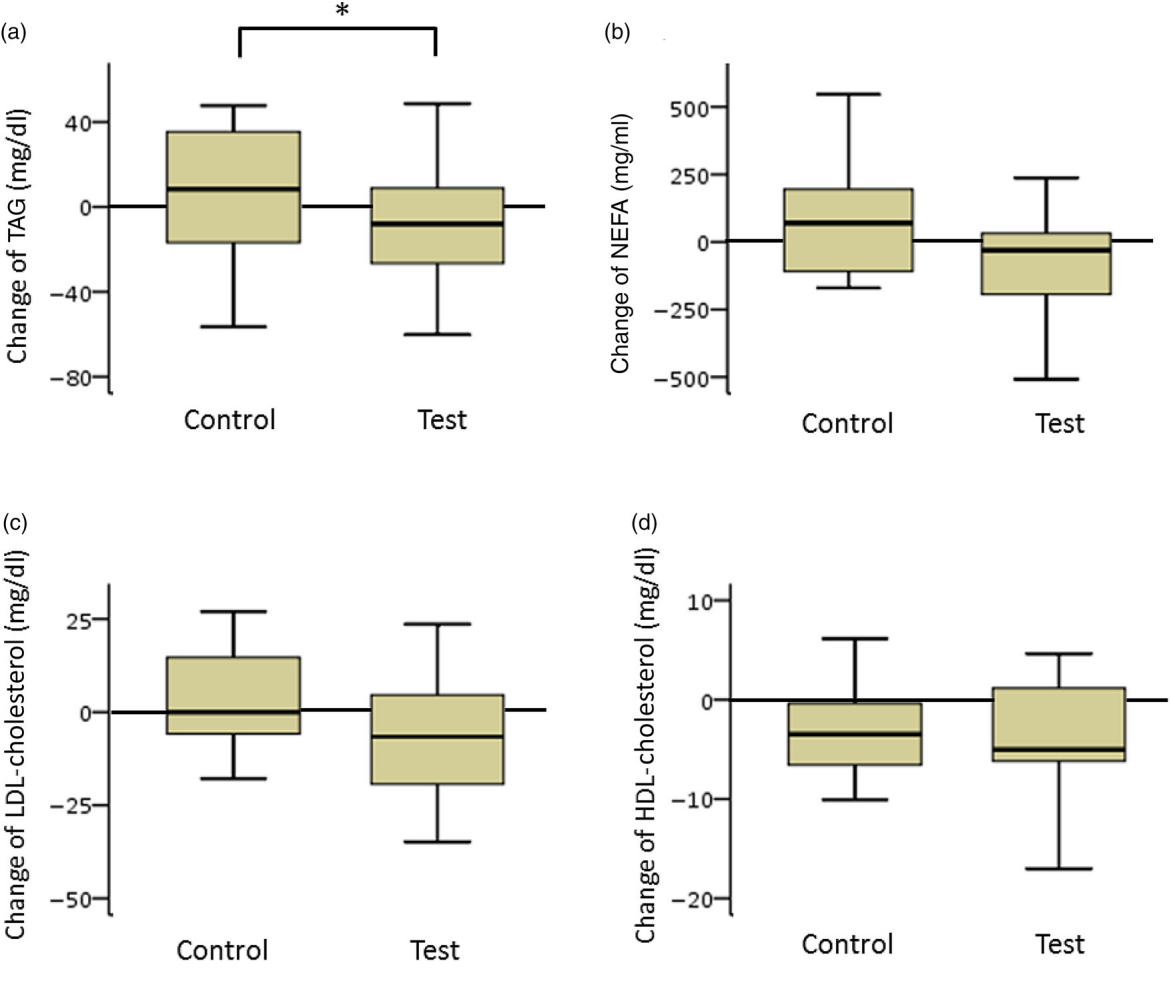
Box plots of net changes in the parameters of lipid metabolism among participants with low insulin sensitivity: (a) TAG; (b) NEFA; (c) LDL-cholesterol; (d) HDL-cholesterol. Whiskers represent the minimum and maximum values, and the boxes represent the lower and upper quartiles. * Significant difference between the test (GLUCODA™) and control groups (*P* < 0·05). To convert TAG from mg/dl to mmol/l, multiply by 0·0113. To convert cholesterol from mg/dl to mmol/l, multiply by 0·0259.

TAG and NEFA levels in the test group decreased considerably. The net changes in TAG levels in the test and control groups were −15 (sd 37) and 10 (sd 33) mg/dl (−0·17 (sd 0·42) and 0·11 (sd 0·37) mmol/l), respectively, and a significant difference was observed between the two groups (*P* < 0·05). The net changes in NEFA levels in the test and control groups were −85 (sd 379) and 45 (sd 286) mEq/l, respectively. The difference between the groups was not statistically significant (Fig. 3).

#### Hepatic functional enzymes (participants with low insulin sensitivity)

**Fig. 4.**
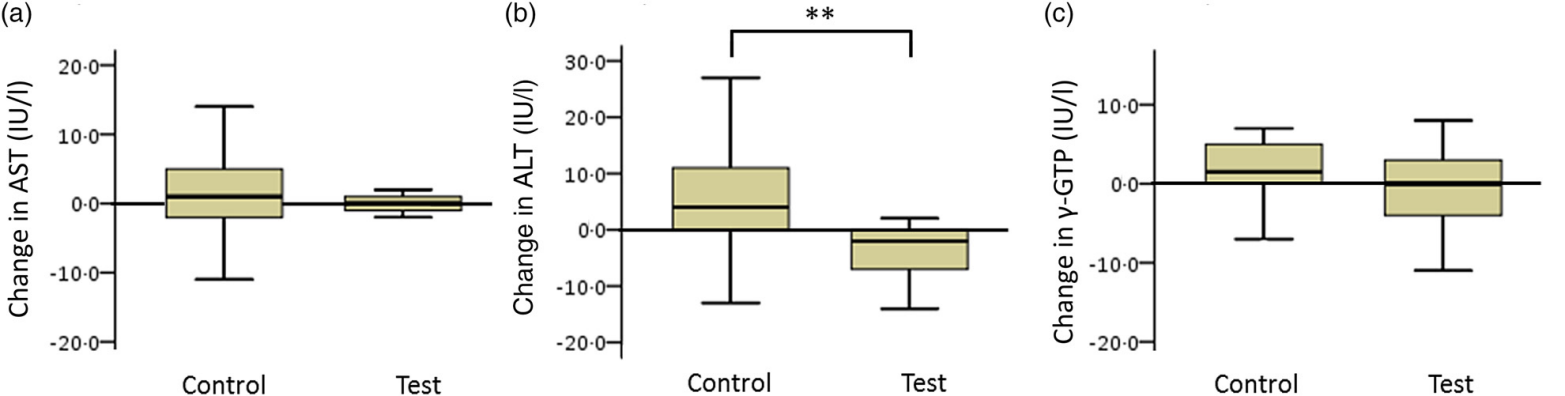
Box plots of net changes in the parameters of hepatic functional enzymes among participants with low insulin sensitivity: (a) aspartate aminotransferase (AST); (b) alanine aminotransferase (ALT); (c) γ-glutamyltransferase (γ-GTP). Whiskers represent the minimum and maximum values, and the boxes represent lower and upper quartiles. ** Significant difference between the test (GLUCODA™) and control groups (*P* < 0·01).

The net changes in ALT levels in the test and control groups were −5·6 (sd 8·4) and 4·6 (sd 18·3) IU/l, respectively, with a statistically significant difference between the two groups (Fig. 4; *P* < 0·01).

#### Body composition (participants with low insulin sensitivity)

The BMI of the test group decreased slightly while the BMI in the control group increased (data not shown). Body composition measurements by dual-energy X-ray absorptiometry revealed a decrease in body fat mass and an increase in lean body mass in the test group (body fat mass, 33·8 (sd 31·4) to 33·6 (sd 35·6) kg; lean body mass, 48·7 (sd 40·3) to 49·0 (sd 42·2) kg). Conversely, in the control group, body fat mass increased and lean body mass decreased (body fat mass, 34·2 (sd 47·3) to 34·7 (sd 48·3) kg; lean body mass, 57·1 (sd 57·2) to 56·9 (sd 52·1) kg). The differences in body fat mass and lean body mass within each group and between the test and control groups were not statistically significant.

## Discussion

The present two studies on MuPI (GLUCODIA^™^) are promising phase II clinical studies that provide a clear scientific rationale for the investigation of specific parameters, such as glucose and liver enzyme chemistry, in future trials. The safety profile of MuPI was evaluated during these two studies and the dose was both safe and well tolerated. Safety parameters remained within clinically acceptable ranges, and all subjects were able to complete the study except for dropouts due to personal circumstances. There were no significant differences in safety parameters between the groups.

In the pre-study, FPG levels in the MuPI 6-g and MuPI 3-g groups decreased after the consumption of the study product (*P* < 0·1, for both) while an increasing trend was observed in the control group. There were significant differences in the net changes in FPG levels between the MuPI 6-g and 3-g groups and the control group (*P* < 0·05, for both). The trend for fasting insulin was similar to that for FPG. The fasting insulin levels in the MuPI 6-g and 3-g groups showed a decreasing trend, while an increasing trend was observed in the control group. HOMA-IR values for both the MuPI 6-g and the 3-g groups decreased, although these changes were also not statistically significant. The majority of the lipid parameters were within the normal range at baseline. However, TAG levels for the MuPI 3-g group were high at baseline. They decreased progressively and, by week 4, these were under 100 mg/dl (1·13 mmol/l). BMI showed a similar trend to FPG and insulin. Based on these results, the intake of 3 g of MuPI was considered the minimum dose required to produce physiological effects.

Results from the main study showed that mung bean protein was effective at significantly reducing insulin concentrations after an 8-week supplementation. A significant decrease in HOMA-IR and increase in adiponectin levels in the test group relative to the control group were observed. Additionally, though it was not significant, a decreasing trend in NEFA in the test group relative to the control group was observed. Insulin resistance has emerged as a major pathophysiological factor in the development and progression of diabetes since it is evident in susceptible individuals at the early stages of diabetes, and particularly type 2 diabetes. Insulin resistance is usually defined as a value greater than the 75th percentile value for non-diabetic subjects according to the WHO^(31)^. Furthermore, assessment of insulin resistance using the HOMA-IR is a key index for the primary prevention of diabetes and is recommended for screening of high-risk groups. Different HOMA-IR cut-off values have been reported^(^^30^^)^. This study examined subjects with a HOMA-IR value over 1·7 in order to evaluate the improvement of consuming GLUCODIA™ on glucose tolerance in subjects with the most mild insulin resistance. The beneficial effects of GLUCODIA™ on glucose tolerance were greater in subjects with low insulin sensitivity. In this study, there were no significant differences between the groups in terms of body fat, fat body mass and lean body mass by body dual-energy X-ray absorptiometry scans^(^^32^^)^. However, decreasing trends in percentage of body fat and fat body mass and an increasing trend in lean body mass were observed among subjects in the MuPI group. Furthermore, plasma adiponectin concentrations significantly increased in the MuPI group. The negative correlation between visceral adiposity and adiponectin levels may be partly explained by the increased secretion of this adiponectin from accumulated visceral fat^(^^33^^)^. Therefore, the decreasing trends in percentage of body fat and fat body mass suggest a reduction in visceral fat. A decrease in body weight and body fat, resulting from a decrease in visceral fat, may be more predictably expected with ingestion of MuPI over a longer period. Blood lipid parameters and hepatic functional enzymes (aspartate aminotransferase, ALT and γ-glutamyltransferase) were found to be within the normal range, except for TAG and ALT. TAG levels at baseline for the MuPI group were slightly above the normal range, although at week 8 TAG levels had decreased, and this trend was particularly noticeable among subjects with low insulin sensitivity (HOMA ≥ 1·7). Thus, there were significant differences in the net changes in TAG levels between the MuPI group and the control group. High ALT has been reported to be an independent risk factor for non-alcoholic fatty liver disease (NAFLD)^(^^34^^)^. NAFLD is known to be associated with diabetes and is an important feature of the metabolic syndrome and insulin resistance^(^^35^^)^. In their study on mice, Watanabe *et al*.^(^^36^^)^ reported that MuPI may play a preventative role in NAFLD by reducing hepatic lipid accumulation. MuPI has been reported to reduce plasma TAG levels by the active suppression of fatty acid synthase by inhibition of sterol regulatory element-binding protein 1 (SREBP-1) expression in rat liver^(^^27^^)^. The results obtained in these studies are in good agreement with the results reported for β-conglycinin^(^^12^^,^^37^^)^, which closely resembles the 8S globulin of MuPI with respect to structural properties^(^^24^^)^. Therefore, 8S globulin and β-conglycinin may have peptides of consensus sequence that exert these beneficial effects. However, Watanabe *et al*.^(^^36^^)^ reported that MuPI may have different gene expression systems from SPI, including β-conglycinin, in *de novo* lipogenesis (DNL)-related gene-expression control. It is known that DNL, fatty acid uptake and fatty acid oxidation are involved in the process of hepatic lipid accumulation^(38)^. Moreover, Watanabe *et al*.^(^^36^^)^ reported that the suppressive effect of MuPI on DNL-related gene expression was not induced by a mixture of amino acids matching the composition of MuPI, and this effect is not related to the amino acid composition of MuPI *per se*. This report postulated the existence of a unique MuPI peptide sequence, distinct from the sequence in soya protein contributing to improvement of the lipid metabolism in the liver.

MuPI (GLUCODIA^™^) is safer than soya protein in terms of being a non-GM organism and there are no reported cases of allergies as in soyabeans. In the present study, it was shown that GLUCODIA^™^ has beneficial effects on the metabolic syndrome and it is a food material that can be expected to have these effects at a smaller amount of intake compared with the intake recommended by the Food and Drug Administration for soya protein.
